# Distinct Regulation of Transmitter Release at the *Drosophila* NMJ by Different Isoforms of *nemy*

**DOI:** 10.1371/journal.pone.0132548

**Published:** 2015-08-03

**Authors:** David Knight, Konstantin G. Iliadi, Natalia Iliadi, Ronit Wilk, Jack Hu, Henry M. Krause, Paul Taylor, Michael F. Moran, Gabrielle L. Boulianne

**Affiliations:** 1 Program in Developmental and Stem Cell Biology, Peter Gilgan Center for Research and Learning, The Hospital for Sick Children, Toronto, M5G 0A4, Canada; 2 Dept of Molecular Genetics, University of Toronto, Toronto, Ontario, M5S 1A8, Canada; 3 Department of Molecular Genetics, The Terrence Donnelly Centre for Cellular and Biomolecular Research, University of Toronto, Toronto, ON, M5S 3E1, Canada; 4 Program in Molecular Structure and Function, The Hospital for Sick Children, Toronto, M5G 1L7, Canada; University of Würzburg, GERMANY

## Abstract

Synaptic transmission is highly plastic and subject to regulation by a wide variety of neuromodulators and neuropeptides. In the present study, we have examined the role of isoforms of the cytochrome b561 homologue called *no extended memory* (*nemy*) in regulation of synaptic strength and plasticity at the neuromuscular junction (NMJ) of third instar larvae in *Drosophila*. Specifically, we generated two independent excisions of *nemy* that differentially affect the expression of *nemy* isoforms. We show that the *nemy* ^45^ excision, which specifically reduces the expression of the longest splice form of *nemy*, leads to an increase in stimulus evoked transmitter release and altered synaptic plasticity at the NMJ. Conversely, the *nemy* ^26.2^ excision, which appears to reduce the expression of all splice forms except the longest splice isoform, shows a reduction in stimulus evoked transmitter release, and enhanced synaptic plasticity. We further show that *nemy* ^45^ mutants have reduced levels of amidated peptides similar to that observed in peptidyl-glycine hydryoxylating mono-oxygenase (PHM) mutants. In contrast, *nemy* ^26.2^ mutants show no defects in peptide amidation but rather display a decrease in Tyramine β hydroxylase activity (TβH). Taken together, these results show non-redundant roles for the different *nemy* isoforms and shed light on the complex regulation of neuromodulators.

## Introduction

The strength of transmitter release is highly plastic and can be modulated both acutely and chronically. The modulation of synaptic strength in response to specific inputs facilitates learned behaviours and allows synapses to synchronize their outputs to specific environmental cues. Synaptic strength may be modulated by the action of various neuromodulators including neuropeptides and biogenic amines such as dopamine, octopamine and serotonin, which exert varied effects on synapses through their respective receptors [1–7]. The modulation of synaptic strength by such neuromodulators facilitates the integration and co-ordination of multiple inputs. In this manner, neuromodulators play an essential role in regulating synaptic plasticity, which is essential for the survival of an organism in an ever-changing environment. It is therefore not surprising that there has been considerable interest in understanding the mechanisms by which neuromodulators regulate synaptic plasticity and behaviour.

We have previously shown that *nemy*, a cytochrome b561 homologue in *Drosophila* is required for aversive olfactory memory [8]. *nemy* is expressed in the larval and adult CNS. In the adult CNS, *nemy* shows strong expression in the antennal lobe and mushroom bodies, structures known to be involved in olfactory learning and memory [8]. In the larval CNS, *nemy* is expressed in several cells in the midline of the ventral nerve cord. Cytochrome b561 homologues maintain a pool of reduced intra-vesicular ascorbic acid by the transfer electrons generated by the oxidation of ascorbic acid in the cytoplasm across secretory vesicle membranes to reduce intra-vesicular semi-dehydroascorbate, producing ascorbic acid (Reviewed in [9]. Ascorbic acid is a necessary co-factor for mono-oxygenases such as Peptidyl-glycine hydroxylating mono-oxygenase (PHM) and Tyramine β hydroxylase TβH [10]. Both PHM and TβH affect a wide variety of behaviours and biological processes in flies.

PHM catalyzes the first step in a two-step reaction that converts a glycine residue at the C-terminal end of neuropeptide precursors into an α-amide [11,12], thus producing an active amidated neuropeptide. It is estimated that as many as 90% of all neuropeptides in *Drosophila* require C-terminal amidation to be biologically active [13]. PHM activity has been directly linked to the regulation of several behaviours and biological processes including developmental transitions during embryonic and larval development [13] and circadian locomotor rhythms [14]. In addition, specific neuropeptide precursor genes such as amnesiac, and dFMRF have been shown to affect behaviours including sleep maintenance [15], learning and memory [16–18], synaptic function and plasticity [19–21]. TβH is a homologue of dopamine β-hydroxylase (DβH) in mammals, which converts dopamine into nor-epinephrine [22]. In flies, TβH converts tyramine to octopamine, which acts as both a neuromodulator and neurohormone, similar to nor-epinenphrine in mammals [22,23]. Octopamine has been shown to affect a wide array of behaviours including egg laying [22], social behaviours [24,25] learning and memory [26] and synaptic function and plasticity [27,28].

Several studies have shown that larval NMJ function is regulated by both neuropeptides and neuromodulators such as octopamine [21,28–30]. It is possible therefore, that mutations in *nemy* may lead to changes in NMJ function. Furthermore, it is possible that *nemy* may exert multiple effects on NMJ function through effects on both neuropeptide amidation and octopamine synthesis. In the present study, we have addressed this question by examining synaptic strength and plasticity in third instar larval NMJs and compared the effects of mutations in *nemy* mutants to those observed in PHM and TβH mutants. We show that the *nemy* gene gives rise to multiple isoforms. We generated independent deletions within the *nemy* gene that differentially affect the expression of different *nemy* isoforms. We show that loss of the longest splice form of *nemy* leads to a significant increase in stimulus evoked transmitter release while loss of the shorter splice forms leads to a decrease in stimulus evoked transmitter release. We also observed significantly different effects on synaptic plasticity in the two *nemy* mutants. We show that the differences in synaptic function observed in the *nemy* mutants may be explained by differences in the effects of these mutations on PHM and TβH activity, suggesting unique roles for the different *nemy* isoforms with regard to PHM and TβH function.

## Materials and Methods

### Fly Stocks

All stocks were raised on standard fly food, with a 12/12 hr light/dark cycle, at 23 ± 1°C and 45%- 50% relative humidity. *w* ^*1118*^ flies that had previously been outcrossed for 10 generations with Canton S were used as the wild-type strain in this study. The *nemy* ^*45*^ excision was previously described [8]. The *nemy* ^*26*.*2*^ excision was generated via imprecise excision of P{EP}^16037^ obtained from the GenExel Inc Stock centre. Both the *nemy* ^*45*^ and *nemy* ^*26*.*2*^ excisions were outcrossed to the cantonized *w* ^1118^ strain for at least 5 generations prior to the start of experiments. The tyramine β-hydroxlase precise (M6) and imprecise (M18) excision mutants were generated by M. Monastirioti (Institute for Molecular Biology and Biotechnology, Heraklion Greece) [22]. Both M6 and M18 lines were outcrossed to Canton S by Sarah Certel (Center for Structural and Functional Neuroscience, University of Montana). The M18 TβH mutants were maintained over a *y*, FM7 balancer chromosome. Experiments were performed on M18 males with a non *y* bristle phenotype. To eliminate any possible gender based variability, male M6 larvae were used as controls. The PHM ^07623^ mutant line in was a gift from P. Taghert (Department of Anatomy and Neurobiology, Washington University, St Louis). PHM^07623^ flies were balanced over a 2^nd^ chromosome balancer with a ubiquitous GFP dominant marker to facilitate genotyping in embryonic and larval stages. To avoid genetic background issues arising from the presence of a balancer chromosome, balanced mutants were crossed to wild-type CS flies, and the non-GFP larvae were selected for experiments.

### Electrophysiology

Larvae were dissected [31] in a haemolymph like solution HL3.1 [32] containing 0.2 mM calcium and pinned to the base of a Sylgard (Dow Corning) coated perfusion bath. The segmental nerves exiting the ventral ganglion were cut and drawn into a glass-stimulating pipette and stimulated with square wave pulses (0.15 ms, 5–15V) to generate action potentials in the motor neurons. Unless otherwise stated, intracellular recordings were obtained from muscle 6 of abdominal segments 3 and 4 with sharp intracellular microelectrodes filled with a 50/50 mix of 3 M potassium chloride and 3 M potassium acetate (20–50 MW). Stimulus evoked excitatory junction potentials (EJPs) and spontaneous miniature EJPs (mEJPs) were recorded using an Axoclamp 2B amplifier and 0.1 LU head stage from Axon Instruments. Analog signals were low-pass filtered at 5 kHz, digitized at 20 kHz by a Powerlab/4SP A/D converter and saved on a computer for later analysis using Mini Analysis software (Synaptosoft). Quantal content was calculated independently for each NMJ by dividing the EJP amplitude by the mean mEJP amplitude.

### RT-PCR

Total RNA was extracted from adult flies and purified using Mini RNA isolation columns from Invitrogen. Isolated RNAs were DNase treated using amplification grade DNase I (Invitrogen). DNase treated RNA was reverse transcribed using a SuperScript II reverse transcriptase kit from Invitrogen and 200–250 ng random hexamers per reaction. cDNA was amplified by PCR using the a single reverse primer that recognizes all *nemy* isoforms (TTCTCACAGCACAGCATTAC) and the following isoform specific forward primers: nemy^RA^, (CATTGAAAGGGTTTTTGAC), nemy^RF^, (GATTAGCAGCACATTCAACC), nemy^RB, RC,RE, RG^ (TTCTCACAGCACAGCATTAC). Note that due to the sequence similarity between nemy transcripts RB, RC, RE, and RG, these transcripts were all amplified in a single PCR reaction. The ribomal protein L32 (RP49) was used as a loading control and was amplified using the following primers (fwd: TTGAAGCTGGAAGGACACAA, rev: AATTCGGATCGATTCCTGTG). PCR reactions were run on a 1% agarose gel, and stained with Red-Safe nucleic acid stain.

### Mass Spectrometry of amidated neuropeptides

The CNS of 50 third instar larvae were dissected and crushed in 40 ul of an ice-cold solution of methanol/water/formic acid (90:9:1) solution, filtered through Millipore spin-down filters (0.22mm, 6000 rpm for 10 min) dried and stored at -20°C. The pellets were resuspended in 5% acetonitrile and 0.1% formic acid. The peptides were separated by LCMS on a reverse phase column (Magic C18, Michrome Biosciences) using a split-free nano chromatography system (EASY-nLC, Proxeon, Odense Denmark). Samples were loaded via a C18 pre-column and analysis was carried out in duplicate. Elution was performed across a gradient from 5% to 30% acetonitrile in 0.1% Trifluoroacetic acid over 50 min. Peptide detection was carried out using a linear ion trap/FTMS hybrid mass spectrometer (LTQ-Orbitrap, Thermo-Fisher, Bremen Germany) operated in a data dependent mode. Peptide MS data was collected at a resolution of 60,000 full-width half maximum. Tandem mass spectra were obtained in parallel in the lineAr ion trap. The tandem mass spectra were compared to the *D*. *melanogaster* database (NCBI) using the Sequest search engine (Thermo-Fisher, San Jose CA). Potential modifications included in the search were amidated C-termini and oxidized methionine residues. The mass accuracy used in the search was 7 ppm for the parent ion and 0.5 Daltons for the fragment ions. Peptide quantitation was carried out using the Xcalibur 2.0 software (Thermo-Fisher, San Jose CA) generated extracted ion current for the monoisotopic isotopic peak of the doubly charged ion. The total area of the extracted peak for each ion was calculated for all samples. All measurements were done in triplicate biological replicates each of which contained two technical repetitions.

### NMJ morphology

To examine the synaptic morphology, we transferred 25 male and 25 female flies to fresh bottles and allowed them 24 hrs to lay eggs. The control and *nemy* mutant third instar larvae from these bottles were dissected to expose the musculature of the body wall in phosphate buffered saline (PBS) then fixed at room temperature in 4% formaldehyde in PBS. Neuromuscular junctions were labeled with FITC-conjugated anti-horse radish peroxidase (1: 400, ICN Biomedicals, CA). Images were captured as a Z-stack using a Leica SP8 confocal microscope. Z-stacks were compressed into a single image and the total number of boutons on muscles 6 and 7 were counted.

### Fluorescent *in situ* hybridization

Fluorescent in situ hybridization (FISH) experiments were performed on Oregon R embryos essentially as described in Wilk et. al [33]. Two RNA probes were used to distinguish the localization of the *nemy* ^RA^ isoform from other *nemy* isoforms. The first probe was chosen to specifically anneal to *nemy* ^RA^ transcripts, while the second probe was designed to hybridize to all *nemy* transcripts. Probes were DIG-labelled for detection with tyramide signal amplification-FISH as described in Wilk et al [33].

The sequence of the primers used to generate the probes were as follows (T7 promoter underlined):

*nemy* ^RA^ Fwd:5’ TTCGTTCGCAGCATTCGTGG 3’

*nemy* ^RA^ Rev: 5’TAATACGACTCACTATAGGGAGTTCGTGTGGCGTG 3’

All transcripts Fwd: 5’ ACTACATCACCAGTGCCATC 3’

All transcripts Rev:

5’ GTAATACGACTCACTATAGGGAGACCACCTTGGTCTTCTGCTCATCTG 3’

### Statistical analysis

Data were analysed statistically using an ANOVA and t-test. All statistics were performed using Excel and Sigmaplot software.

## Results

**Fig 1:**
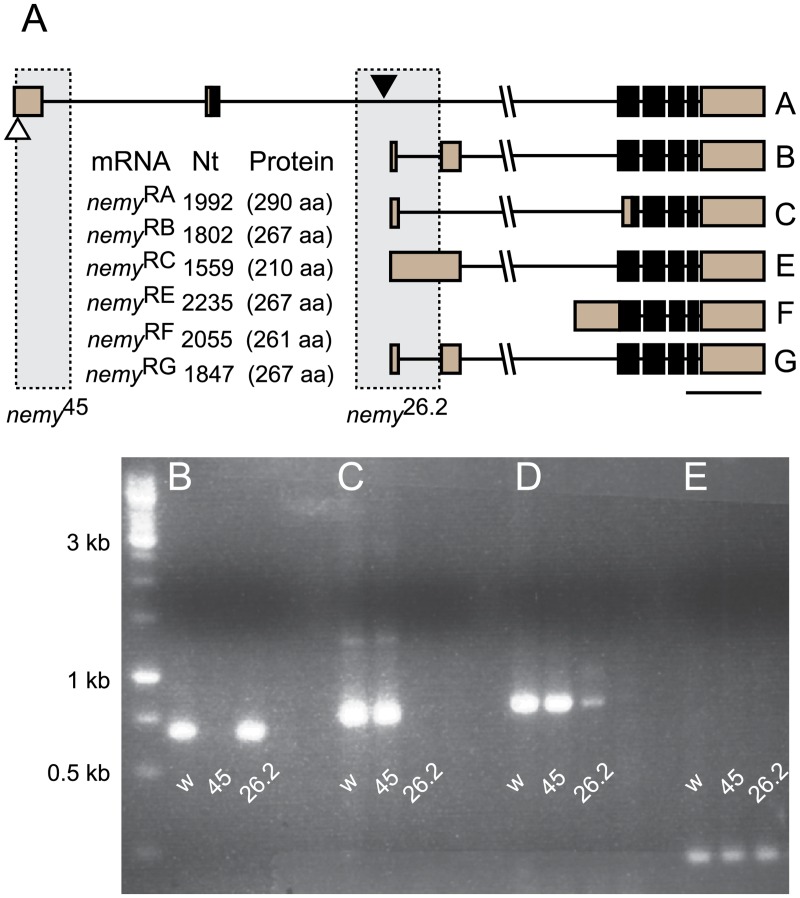
Excision mutants have different effects on *nemy* expression. **A.** Schematic of the genomic organization of nemy. The P{LacW}^153^ P element used to generate the *nemy* ^45^ excision is represented by an open triangle, while the P{EP}^16037^ P-element used to generate the *nemy* ^26.2^ excision is shaded in black. The *nemy* ^45^ and *nemy* ^26.2^ excisions are indicated by shaded boxes. Scale bar indicates 1 Kb. **B-E.** RT-PCR of *nemy* isoforms in control (w), *nemy* ^45^ and *nemy* ^26.2^ excisions. **B.** The *nemy* ^RA^ isoform is absent in *nemy* ^45^ mutants, but not affected in *nemy* ^26.2^ mutants. **C.** Conversely, isoforms B, E and G are present at control levels in *nemy* ^45^ mutants, but absent in *nemy* ^26.2^ mutants. Note, we were unable to detect a band that corresponded to *nemy* isoform C. **D.** The *nemy* isoform F is expressed at control levels in *nemy* ^45^ mutants, but significantly reduced in *nemy* ^26.2^ mutants. **E.** The house keeping gene RP49 was expressed at comparable levels in all genotypes and no signal was detected in ‘no transcript controls’ (NTC).

*nemy* was originally generated in a forward genetic screen for mutants affecting courtship memory [34]. A more thorough characterization showed that in addition to defects in courtship memory, *nemy* mutants also have a defect in middle-term olfactory memory [8]. The mutant used in both of these studies was a P{LacW}^153^ insertion in the 1^st^ exon of *nemy* which specifically affects the expression of the longest splice form of the gene. However, Northern Blot analysis showed at least 4 distinct splice forms [8] and several shorter splice forms of *nemy* are also predicted in flybase (www.flybase.org). To determine whether the different splice forms of *nemy* have distinct functions we have generated two novel mutations within the *nemy* locus, named *nemy* ^*45*^ and *nemy* ^*26*.*2*^ by excision of two independent transposable elements, P{LacW}^153^ and P{EP}^GE16057^ respectively (Fig 1). Both excisions are homozygous viable and breakpoints were confirmed by PCR and subsequent sequencing analysis. Interestingly, the *nemy* ^45^ and *nemy* ^*26*.*2*^ excisions exert different effects on the expression of *nemy* isoforms. Using primers specific for each transcript (see Materials and Methods), we examined the expression of the different *nemy* transcripts by RT-PCR. As shown in Fig 1, expression of the *nemy* ^RA^ transcript is significantly reduced in *nemy* ^45^ mutants, but is not affected in *nemy* ^*26*.*2*^ mutants. Conversely, expression of the remaining *nemy* transcripts is significantly reduced in *nemy* ^*26*.*2*^ mutants, but not affected in *nemy* ^45^ mutants.

### Basal transmitter release and synaptic plasticity are impaired in *nemy* mutants

**Fig 2:**
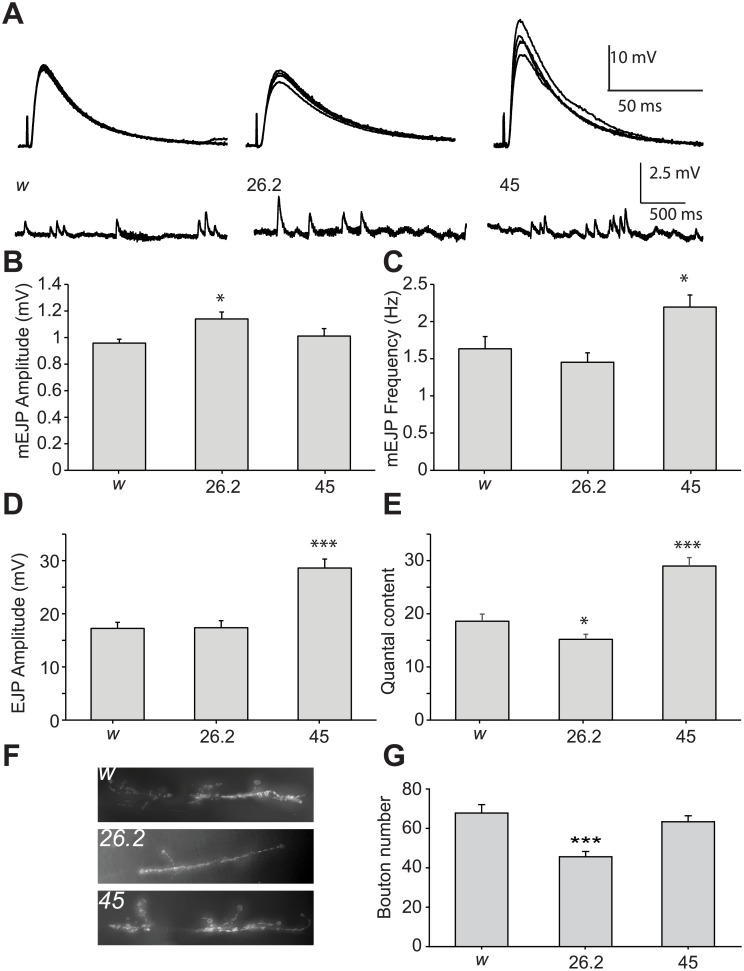
Basal transmitter release is altered in *nemy* mutants. **A**. Example stimulus evoked EJPs and spontaneous mEJPs. **B-C.** The amplitude (B) and frequency (C) of miniature EJPs in control (w) and *nemy* mutants. **D**. Stimulus evoked EJP amplitude in control (*w*) and *nemy* mutants. **E.** Quantal content in control (*w*) and *nemy* mutants. **F.** Example NMJs from control (*w*) and *nemy* mutant third instar larvae. Synaptic boutons are stained with a FITC-conjugated antibody raised against horse-radish peroxidise. **G.** Quantification of the number of synaptic boutons in control and *nemy* mutant NMJs. * indicates a significant differences of P < 0.05, ** indicates P < 0.01 and *** indicates P < 0.001. Statistical differences were calculated by ANOVA followed by multiple comparisons using the Student-Newman-Keuls method.

Both the *nemy* ^*45*^ and *nemy* ^*26*.*2*^ mutants were outcrossed to cantonized *w* ^*1118*^ flies for five generations prior to the commencement of experiments to avoid differences in the genetic background of the mutants. To assess whether either the *nemy* ^45^ or *nemy* ^*26*.*2*^ mutations affect synaptic transmission, we examined stimulus evoked and spontaneous transmitter release at the neuromuscular junctions of third instar larvae. We first examined the effect of the *nemy* ^*45*^ and *nemy* ^*26*.*2*^ excisions on spontaneously occurring miniature excitatory junction potentials (mEJPs) in muscle 6 of abdominal segments 3 and 4. Interestingly, we observed different effects of the *nemy* ^*45*^ and *nemy* ^*26*.*2*^ excisions on both the amplitude and frequency of spontaneous transmitter release compared to the *w* ^*1118*^ control (Fig 2). *nemy* ^*26*.*2*^ mutants showed a small but significant increase in mEJP amplitude compared to control without any significant change in the frequency of mEJPs. In contrast, *nemy* ^*45*^ mutants showed no significant change in mEJP amplitude, but did show a significant increase in the frequency of mEJPs. (Table 1, Fig 2B and 2C). Differences in mEJP amplitude are often equated with a change in the density of postsynaptic receptors, while changes in the frequency of mEJPs are exclusively regulated by the presynaptic neuron [35,36]. These observations of changes in mEJP amplitude and frequency may therefore indicate unique roles for the different isoforms of *nemy* in both the presynaptic and postsynaptic cells of the NMJ.

**Table 1:** Basal transmitter release and synaptic plasticity in control (*w* ^1118^) and nemy mutant flies.

	*w* ^*1118*^	*nemy* ^*26*.*2*^	*nemy* ^*45*^
mEJP Amplitude (mV)	0.95 +/- 0.03	1.14 +/- 0.05*	1.01 +/- 0.05
mEJP Frequency (Hz)	1.63 +/- 0.16	1.45 +/- 0.13	2.19 +/- 0.16*
EJP Amplitude (mV)	17.26 +/- 1.13	17.36 +/- 133	28.6 +/- 1.68***
Quantal Content	18.61 +/- 1.32	15.19 +/- 0.93*	28.99 +/- 1.6***
Synaptic Boutons	67.8 +/- 4.3	45.6 +/- 2.7***	63.4 +/- 3.0
Paired Pulse Ratio			
20ms ISI	0.99 +/- 0.0.06	1.21 +/- 0.11	0.84 +/- 0.04
100 ms ISI	1.17 +/- 0.03	1.26 +/- 0.06*	1.06 +/- 0.02*
500 ms ISI	67.8 +/- 4.3	1.08 +/- 0.03	0.97 +/- 0.01
PTP ratio	1.5 +/- 0.07	1.8 +/- 0.13	1.4 +/- 0.1

* indicates a significant difference (P < 0.05) relative to *w* ^1118^,

*** indicates a significant difference (P < 0.001) relative to *w* ^1118^.

Statistical differences determined by ANOVA followed by multiple comparisons using the Student-Newman-Keuls method.

We next examined the effect of *nemy* mutations on stimulus evoked transmitter release. Single pulses were delivered to cut motor neurons at a frequency of 0.2 Hz and the stimulus evoked EJPs were recorded from muscle 6 in abdominal segments 3 and 4. Compared to the control (*w* ^*1118*^), we observed a small decrease in stimulus evoked EJP amplitude in *nemy* ^*26*.*2*^ mutants (Table 1, Fig 2D). Combined with the increased amplitude of mEJPs in these mutants, this amounted to a significant decrease in quantal content of transmitter release from *nemy* ^*26*.*2*^ mutant NMJs (Table 1, Fig 2E). By contrast, both stimulus evoked EJP amplitude and Quantal content were significantly increased in *nemy* ^*45*^ mutants compared to control (Table 1, Fig 2D and 2E).

Changes in transmitter release from the NMJ are often coupled with morphological changes including altered axon branching and changes in the number of synaptic boutons. To determine whether the observed changes in transmitter release observed in our *nemy* mutants also affected NMJ morphology, NMJs of third instar larvae were stained with FITC conjugated anti-horse radish peroxidise (FITC-HRP) that labels all post-mitotic neurons in *Drosophila* [37]. We observed a small but significant decrease in the number of synaptic boutons in *nemy* ^*26*.*2*^ mutants, but no significant change in bouton number in *nemy* ^*45*^ mutants (Table 1, Fig 2F and 2G). Taken together, these results indicate that *nemy* is required for proper function at the larval NMJ. Furthermore, these data suggest that different isoforms of *nemy* have distinct effects on NMJ function and morphology.

**Fig 3:**
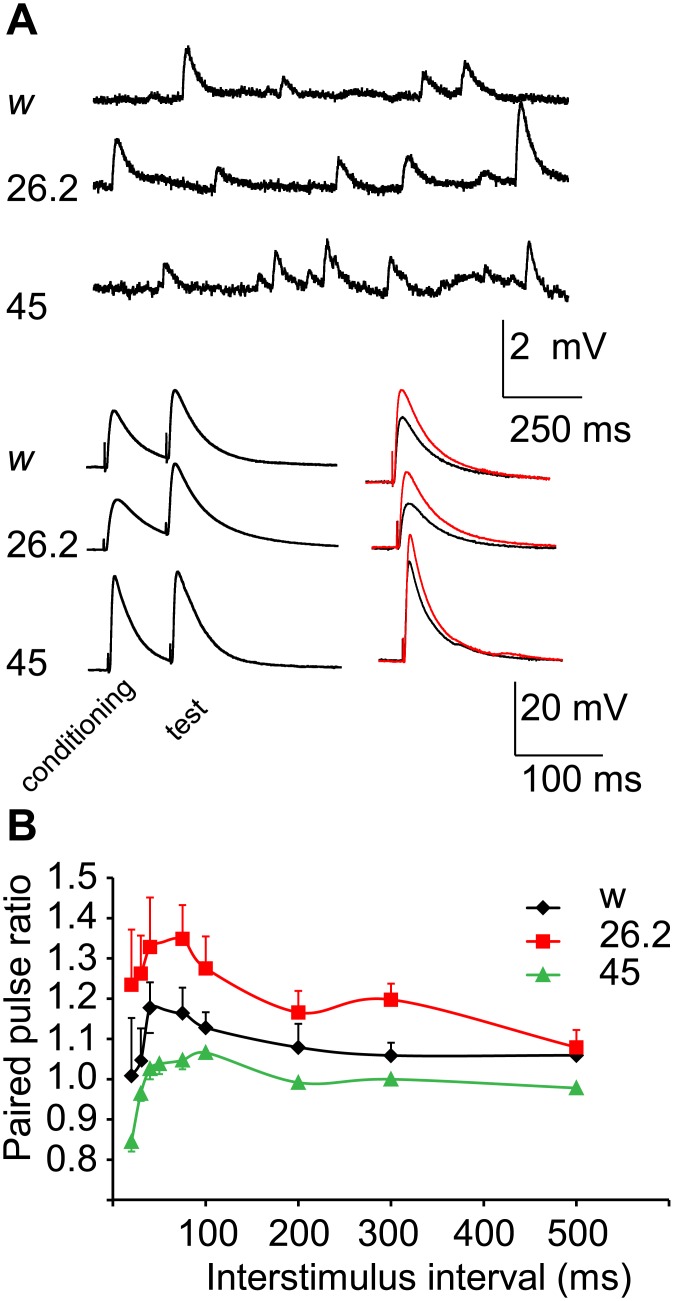
Synaptic plasticity is altered in *nemy* mutants. **A.** Example traces showing spontaneous mEJPs and stimulus evoked EJPS in control (w) and nemy mutants. Stimulus evoked EJPs were delivered in pairs and the paired pulse ratio is calculated as the ratio of the test pulse (red) relative to the conditioning pulse (conditioning). **B.** Paired pulse ratio across multiple interstimulus intervals (ISI). Control (w, black) larvae showed mild paired pulse potentiation at short ISI. This paired pulse potentiation was enhanced in *nemy* ^26.2^ mutants (red) while *nemy* ^45^ mutants (green) show paired pulse depression at short ISI.

Given the observed defects in basal transmitter release, we next asked whether *nemy* mutants show impaired synaptic plasticity. We first looked at paired pulse plasticity by delivering pairs of stimuli with varying inter-stimulus intervals and comparing the amplitude of the second (test) EJP relative to the amplitude of the first (conditioning) EJP across genotypes (Fig 3B). At short inter-stimulus intervals, the test pulse showed moderate facilitation in control preparations under the conditions used (0.2 mM extracellular calcium). *nemy* ^*26*.*2*^ mutant NMJs showed a greater facilitation at short inter-stimulus intervals than control NMJs (Table 1, Fig 3C). By contrast *nemy* ^*45*^ mutant NMJs showed a decrease in paired pulse facilitation, and even showed paired pulse depression at the shorter inter-stimulus intervals (less than 50 ms). Paired-pulse plasticity is correlated with transmitter release probability [38]. The increased paired-pulse facilitation observed in *nemy* ^*26*.*2*^ mutants suggests a decrease in the underlying probability of transmitter release, while the reduced facilitation and depression observed in *nemy* ^*45*^ mutants suggests an increase in transmitter release probability. These results are therefore consistent with the observations of decreased and increased Quantal content observed in *nemy* ^*26*.*2*^ and *nemy* ^*45*^ mutants, respectively.

We also examined a different form of synaptic plasticity known as paired pulse potentiation. Previous work has shown that the amplitude of EJPs are temporarily facilitated after a short (30s) tetanus (10 Hz stimulation) is applied [39,40]. We observed an approximately 50% increase in the amplitude of EJPs in control preparations following 30s of 10 Hz stimulation. The post-tetanic-potentiation ratio (calculated as the amplitude of the post-tetanic stimulus evoked EJPs relative to the amplitude of pre-tetanic stimulus evoked EJPs) was significantly higher in *nemy* ^*26*.*2*^ mutant NMJs, but was not significantly different in *nemy* ^*45*^ mutant NMJs (Table 1). Differences in post-tetanic potentiation may reflect a difference in the size of the reserve pool of vesicles or in the ability to mobilize the reserve pools of vesicles, [41–43] suggesting that the reserve pool of vesicles in *nemy* ^*26*.*2*^ mutants may be larger and/or more mobile, despite the decrease in transmitter release probability observed during basal transmitter release.

### *nemy* mutants differentially affect PHM and TβH activity

The physiology data suggests that different *nemy* transcripts have antagonistic effects on NMJ function. The primary role of *nemy* as a cytochrome b561 homologue is to maintain a pool of reduced ascorbic acid in the lumen of secretory vesicles [9]. This pool of reduced ascorbic acid then serves as a co-factor for mono-oxygenase enzymes such as peptidyl-glycine hydroxylating mono-oxygenase (PHM) and tyramine β-hydroxylase (TβH) [10]. PHM performs the rate-limiting step in the C-terminal amidation of neuropeptide precursors while TβH catalyses the conversion of tyramine into octopamine. Octopamine and many neuropeptides act as neuromodulators and have been shown to affect a wide range of behaviours in flies. Previous work has shown that loss of TβH function leads to decreased transmitter release and a reduction in the number of synaptic boutons [27]. Interestingly, these larval NMJ phenotypes are strikingly similar to those observed in the present study in *nemy* ^26.2^ mutants. However, the effect of PHM mutations on NMJ function has not been characterized possibly due to the early larval lethality of homozygous PHM mutants [13]. In mice, PHM function is encoded by the bi-functional gene peptidylglycine a-mono-oxygenase (PAM) [11]. Similar to PHM mutants in flies, homozygous PAM mutants die during development [44]. However, heterozygous animals are viable but still show significant abnormalities related to reduced peptide amidation including impaired synaptic transmission [44].

**Fig 4:**
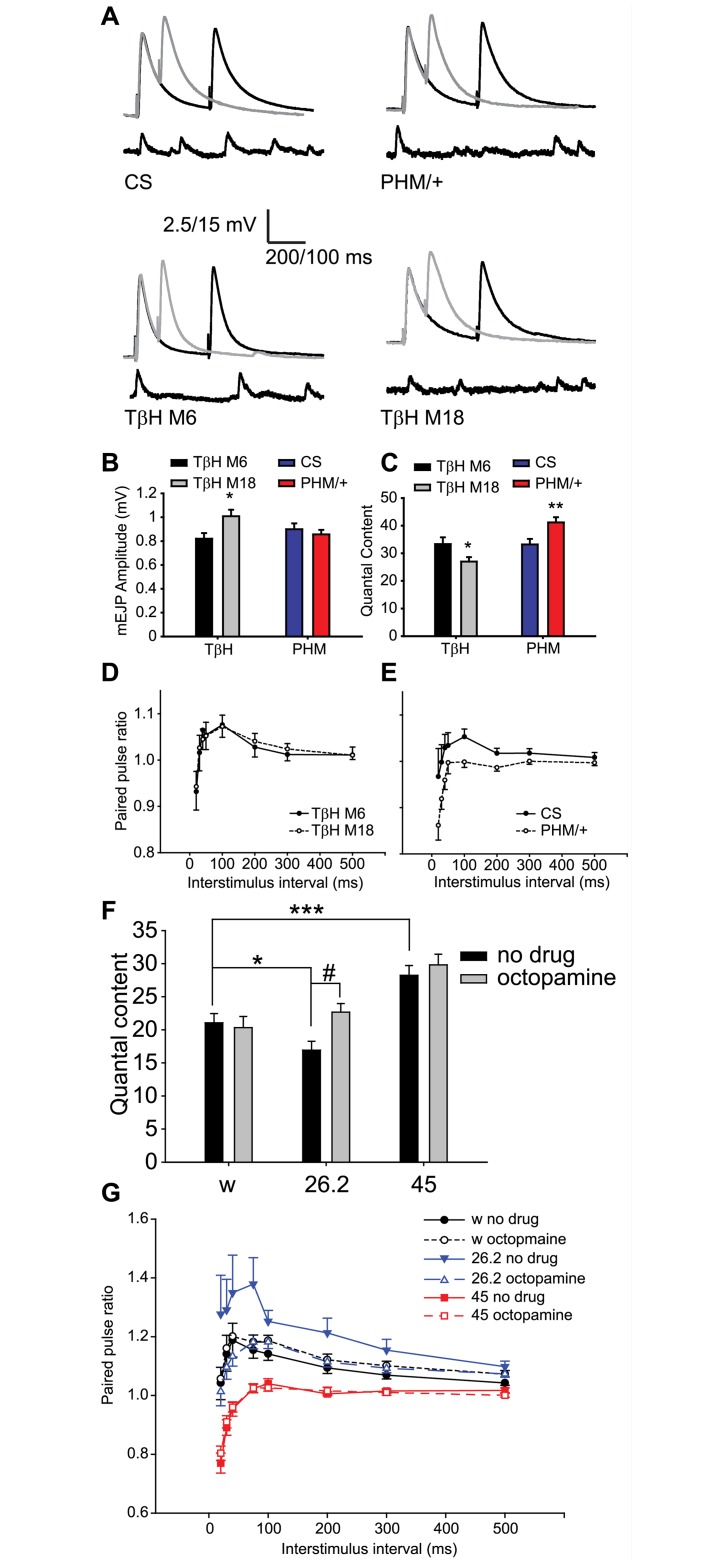
TβH and PHM mutants show similar NMJ phenotypes to *nemy* ^26.2^ and *nemy* ^45^ mutants. **A**. Example traces showing stimulus evoked EJPs and spontaneous mEJPs. **B-C.** mEJP amplitude (B) and Quantal Content (C) in TβH and PHM mutants and their respective controls. TβH M18 imprecise excision mutants were compared to TβH M6 precise excision controls. PHM^P07623^/+ heterozygous mutants (PHM/+) were compared to CS wildtype flies. TβH mutants showed an increase in mEJP amplitude and decrease in Quantal content, while PHM/+ mutants showed an increase in Quantal content, but no change in mEJP amplitude. **D-E.** Paired pulse plasticity in TβH (D) and PHM (E) mutants and the appropriate controls. Paired pulse plasticity in TβH M18 mutants was not significantly different to that in TβH M6 controls. Paired pulse plasticity in PHM/+ mutants was reduced compared to CS controls. **F.** Quantal content in control (*w*) and *nemy* mutants maintained on regular food (Black bars), or on food supplemented with 10 mg/ml Octopamine (grey bars). On regular food, *nemy* ^26.2^ mutants showed a decrease in Quantal content while *nemy* ^45^ mutants showed an increase in Quantal content. Transferring larvae to octopamine supplemented food had no effect on the Quantal content of either control or *nemy* ^45^ animals, but significantly increased the Quantal content of *nemy* ^26.2^ mutants. **G.** Paired pulse plasticity in control and *nemy* mutants maintained on regular food (no drug) or food supplemented with 10 mg/ml octopamine (octopamine). The paired pulse ratio was not affected in either control (Black symbols) or *nemy* ^45^ mutants (red symbols) on an octopamine supplemented diet (compare open symbols to closed symbols). However, the paired pulse ratio was reduced to control levels in *nemy* ^26.2^ mutants (blue symbols) on an octopamine supplemented diet (compare solid inverted triangles in *nemy* ^26.2^ mutants on regular food with open triangles of *nemy* ^26.2^ mutants on octopamine supplemented food). * indicates P < 0.05 relative to the genetic control. ** indicates P < 0.01 relative to the genetic control. *** indicates P < 0.001 relative to the genetic control. # indicates P < 0.05 relative to the pharmacological control (regular food). Statistical differences were determined by ANOVA followed by multiple comparisons using the Student-Newman-Keuls method.

To determine whether the defects in transmitter release observed in *nemy* mutants may reflect changes in either PHM or TβH activity, we first examined transmitter release and synaptic plasticity in TβH mutants and PHM^P07623^ hypomorphic mutants. Male TβH M18 mutants [22] were compared to male M6 precise excision controls (see Materials and Methods for details). Due to the early lethality of homozygous PHM^07623^ hypomorphic mutants, we compared transmitter release in heterozygous PHM mutants to CantonS controls (see Materials and Methods for details). Consistent with previously published results, we observed a decrease in stimulus evoked transmitter release from the NMJs of TβH M18 mutants relative to the M6 control (Table 2, Fig 4C). In our hands, we also observed an increase in the amplitude of spontaneous mEJPs in TβH M18 mutants relative to M6 control (Table 2, Fig 4B). By contrast, PHM heterozygous mutants showed a marked increase in stimulus evoked transmitter release relative to control (Table 2, Fig 4C), but did not show any significant difference in the amplitude of the spontaneous mEJPs. These defects in NMJ function observed in TβH and PHM mutants are strikingly similar to those observed in *nemy* ^26.2^ mutants and *nemy* ^45^ mutants respectively. To further compare the similarities between TβH or PHM mutants and our *nemy* mutants, we next examined the effect of mutations in TβH and PHM on paired pulse plasticity. We observed a significant decrease in paired pulse plasticity in heterozygous PHM mutants relative to the control (Table 2, Fig 4E), but did not see any significant differences in paired pulse plasticity in TβH mutants (Table 2, Fig 4D). The observation of a decrease in paired pulse plasticity in PHM mutants is consistent with our observations in *nemy* ^45^ mutants, and supports the hypothesis that *nemy* ^45^ mutants exert an effect on NMJ function via reduced PHM activity. The lack of any difference in paired pulse plasticity in the TβH mutants however was not consistent with *nemy* ^26.2^ mutants, suggesting that the defects in NMJ function observed in *nemy* ^26.2^ mutants may be only partially due to changes in TβH function.

**Table 2:** Basal transmitter release and synaptic plasticity in TβH and PHM mutants.

	TβH M6	TβH M18	CS	PHM^P07623^/+
mEJP Amplitude (mV)	0.82 +/- 0.05	1.00 +/- 0.05*	0.89 +/- 0.05	0.86 +/- 0.04
Quantal Content	33.3 +/- 2.4	26.70 +/- 1.7*	33.2 +/- 2.0	41.2 +/- 1.9##
Paired Pulse Ratio				
20ms ISI	0.93 +/- 0.04	0.94 +/- 0.03	0.97 +/- 0.06	0.86 +/- 0.03#
100 ms ISI	1.1 +/- 0.03	1.07 +/- 0.03	1.0 +/- 0.02	0.95 +/- 0.01#
500 ms ISI	1.0 +/- 0.02	1.0 +/- 0.01	1.0 +/- 0.01	0.99 +/- 0.01

* indicates a significant difference (P < 0.05) relative to the TbH M6 precise excision control.

^#^ indicates a significant differences (P < 0.05) relative to the CS control and ## indicates a significant differences (P < 0.01) relative to the CS control.

Statistical differences determined by ANOVA followed by multiple comparisons using the Student-Newman-Keuls method.

Studies of TβH mutants have shown that defects in egg laying and locomotion can be rescued by feeding octopamine to the animals [22,45,46]. We hypothesized that if the defects observed in *nemy* ^26.2^ mutants are due, at least in part, to a reduction in TβH activity, we should be able to rescue this by feeding octopamine to larvae. Furthermore, the increased transmitter release observed in *nemy* ^45^ mutants may be partially masked by a concomitant reduction in TβH activity in these mutants. To test these possibilities, control and mutant larvae were transferred to food containing 10 mg/ml octopamine 24–36 hours prior to experiments. We then compared NMJ function in these animals to larvae that were maintained on regular food.

Consistent with our earlier results, *nemy* ^26.2^ mutant larvae maintained on regular food showed reduced stimulus evoked transmitter release and increased altered paired pulse plasticity while *nemy* ^*45*^ mutants maintained on regular food showed increased stimulus evoked transmitter release and altered paired pulse plasticity (Table 3, Fig 4F and 4G). Control larvae that were fed octopamine showed no significant differences in stimulus evoked transmitter release or paired pulse plasticity relative to larvae maintained on regular food (Table 3, Fig 4F and 4G). Similarly, *nemy* ^45^ mutants did not show any change in stimulus evoked transmitter release or synaptic plasticity following octopamine feeding. These results suggest that dietary derived octopamine is not able to exert a lasting effect in control and *nemy* ^45^ mutant larvae. However, *nemy* ^26.2^ mutants fed octopamine showed a significant increase in stimulus evoked transmitter release and a concomitant decrease in paired pulse plasticity compared to larvae maintained on regular food (Table 3, Fig 4F and 4G). Interestingly, stimulus evoked transmitter release and synaptic plasticity in octopamine fed *nemy* ^26.2^ mutant larvae was not significantly different to that observed in control larvae maintained on regular food, suggesting that supplying a source of octopamine to *nemy* ^26.2^ mutant larvae is sufficient to rescue the defects in NMJ function. Furthermore, since the effect of dietary octopamine was specific to *nemy* ^26.2^ mutants, these results suggest that the rescue of synaptic function in octopamine fed *nemy* ^26.2^ mutants must result from regulated release of octopamine from secretory vesicles.

**Table 3:** Effect of octopamine diet on basal transmitter release and paired pulse plasticity.

	*w* ^*1118*^		*nemy* ^*26*.*2*^		*nemy* ^*45*^	
	- Oct	+ Oct	- Oct	+ Oct	- Oct	+ Oct
mEJP Amplitude (mV)	1.14 +/-0.06	1.23 +/- 0.07	1.41 +/- 0.08 *	1.28 +/- 0.06	1.38 +/- 0.09 *	1.39 +/- 0.09
Quantal Content	21.1 +/- 1.4	20.3 +/- 1.7	16.9 +/-1.4 *	22.7 +/- 1.3 #	28.2 +/-1.5 ***	29.8 +/- 1.6
Paired Pulse Ratio						
- 20ms ISI	1.04 +/- 0.06	1.05 +/-0.04	1.27 +/- 0.13	1.01 +/- 0.05 #	0.77 +/- 0.03 ***	0.8 +/- 0.02
- 100 ms ISI	1.14 +/- 0.02	1.18 +/- 0.06	1.25 +/- 0.04 *	1.18 +/- 0.02 #	1.04 +/- 0.02 ***	1.02 +/- 0.01
- 500 ms ISI	1.04 +/- 0.01	1.07 +/- 0.02	1.1 +/- 0.02 *	1.07 +/- 0.01	1.02 +/- 0.01 *	1.00 +/- 0.01

* indicates a significant difference (P < 0.05) relative to the *w* ^1118^ fed on control diet (-Oct).

*** indicates a significant difference (P < 0.001) relative to *w* ^1118^ fed on control diet (-Oct).

^#^ indicates a significant difference (P < 0.05) relative to the same genotype fed on control diet (-Oct). Statistical differences determined by ANOVA followed by multiple pairwise comparisons using the Student-NewmanK-Keuls method. See Fig 4 for graphical representation of data.

**Fig 5:**
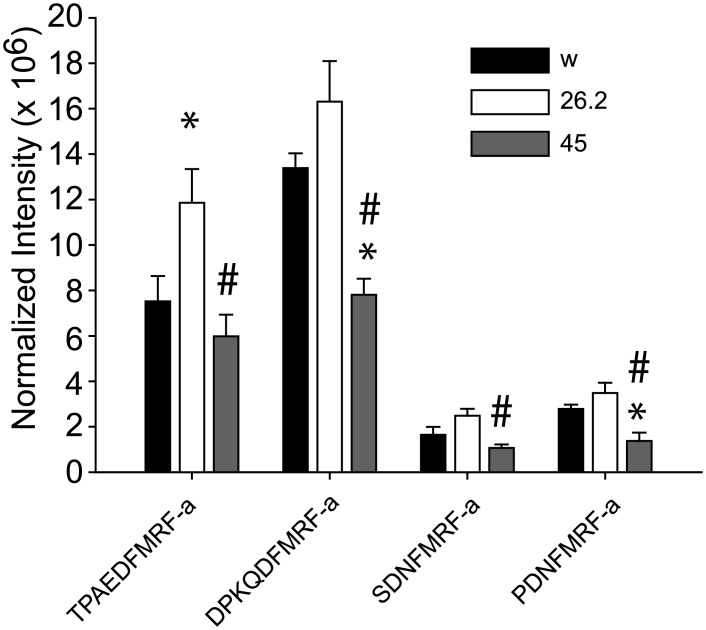
Peptide amidation in control and *nemy* mutants. Mass Spectrometry analysis of peptide amidation in control and *nemy* mutants. The intensity of amidated neuropeptides was normalized against the intensity of non-amidated peptides in the same sample. We observed an increase in the amidation of neuropeptides in *nemy* ^26.2^ mutants, and a decrease in the amidation of neuropeptides in *nemy* ^45^ mutants. The levels of amidated neuropeptides were consistently higher in *nemy* ^26.2^ mutants relative to *nemy* ^45^ mutants. * indicates a significant difference (P < 0.05) compared with *w* ^1118^ and # indicates a significant difference (P < 0.05) compared with *nemy* ^26.2^ mutants.

Finally, to confirm an effect of the *nemy* mutations on PHM activity, we measured the level of amidated neuropeptides in control and *nemy* mutants. Previous work showed that peptide amidation levels were reduced in *nemy* ^45^ mutants [8], however peptide amidation in *nemy* ^26.2^ mutants was not examined. As in the previous study, we used the amidation of the FMRF family of neuropeptides derived from the dFMRF precursor [8] as a measure of peptide amidation. The ANOVA analysis did not reveal any differences (F_2,12_ = 3.44, *p* = .066) between genotypes in the level of a nonamidated peptide (WFGDVNQKPI) that served as an internal control. Thus the intensity of amidated neuropeptides was normalized against the intensity of non-amidated peptides in the same sample. We observed significant differences in neuropeptide amidation in *nemy* ^45^ and *nemy* ^26.2^ mutants (Fig 5). Consistent with our previous work, we saw reduced amidation of the FMRFamide neuropeptides in *nemy* ^45^ mutants. We observed a significant reduction in the levels of both TPAEDFMRFamide (P < 0.05) and PDNFMRFamide (P < 0.005) in *nemy* ^45^ mutants. We also compared the level of amidation between *nemy* mutants and another wild-type *Canton S* control line (Fig 1 in S1 File). Although this line is less appropriate as a genetic background control than cantonised *w* ^1118^, it is nonetheless relevant since all our lines were converted to a *Canton S* background. Moreover, the cantonised *w* ^1118^ control line does not differ from *Canton S* in the levels of amidation for all four FMRFa-like peptides (S1 File). The ANOVA did not find any differences between *Canton S*, *nemy* ^26.2^ and *nemy* ^45^ mutant lines in the level of non-amidated peptides (F_2,12_ = 1.32, *p* = .304) (Fig 1A in S1 File). Similarly, comparisons between genotypes for FMRFa-like peptides revealed that *nemy* ^26.2^ mutants do not differ from *Canton S* whereas *nemy* ^45^ mutants show significant decreases in the amidation for all four FMRFa-like peptides (Fig 1B in S1 File). These results suggest that PHM activity is differentially affected by different *nemy* isoforms.

### *nemy* isoforms show distinct localization

**Fig 6:**
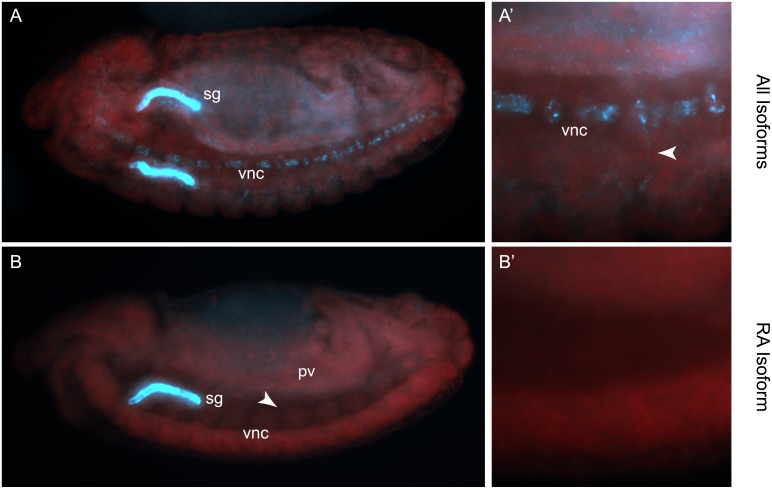
Fluorescent in situ hybridization of *nemy* transcripts. **A** and **A’**. Lateral/ventral view of a stage 15 embryo showing *in situ* hybridization to the salivary gland (sg) and ventral nerve chord (vnc) of a probe that recognizes all *nemy* transcripts. **A’** shows a higher magnification of **A**, arrowhead points to an axon. **B** and **B’**. Lateral/ventral view of a stage 15 embryo showing *in situ* hybridization to the salivary gland (sg) and ventral nerve cord (vnc) of a probe that specifically recognizes the *nemy* ^*RA*^ transcript. **B’** shows a higher magnification of B.

The above results suggest the different *nemy* isoforms differentially affect PHM and TβH activity. We hypothesized that this likely arises from differences in the cellular or sub-cellular localization of the different *nemy* isoforms. Using an antibody that detects all isoforms of Nemy, we have previously shown that Nemy is expressed in both the larval and adult brain and can be detected both pre-and post-synaptically at NMJs of third instar larvae (Figs 1–3 in S1 File). Lacking an antibody capable of distinguishing between individual isoforms of Nemy, we utilized fluorescent *in situ* hybridization (FISH) using two probes against *nemy*, one that specifically detects the longest splice form and a second probe that recognizes all *nemy* isoforms. Since *nemy* expression is highest during embryonic and pupal stages [47], we focussed on the expression of *nemy* transctipts in embryos. As seen in Fig 6, using a probe that recognizes all *nemy* transcripts, we detected *nemy* expression in the embryonic CNS and ventral nerve cord, we also observed fluorescence in processes exiting the ventral nerve cord that may be peripheral axons. In contrast, we were unable to detect expression of the *nemy* ^A^ transcript in the ventral nerve cord indicating that the different transcripts of *nemy* are differentially localized.

## Discussion

The modulation of synaptic strength and plasticity by neuromodulators facilitates learned behaviours and allows synapses to synchronize their outputs to specific environmental cues. The *Drosophila* NMJ has served as a useful model for examining genetic and pharmacological factors that regulate synaptic strength and plasticity for over 30 years. In the present study, we have examined the role of the cytochrome b561 homologue *nemy* on synaptic function and plasticity. We found that *nemy* isoforms differentially affect PHM and TβH function and may therefore indirectly influence some, or all, of the synapses that are modulated by amidated neuropeptides or octopamine.

Since the *nemy* ^45^ excision specifically affects the expression of a single splice form (*nemy* ^A^) while the *nemy* ^26.2^ excisions affects the expression of several splice forms (*nemy* ^B^, *nemy* ^E^ and *nemy* ^G^), we considered the possibility that differences in synaptic function of these observed mutants may reflect differences in the total level of *nemy* expression. However, if *nemy* transcripts were functionally redundant, and the phenotype was a reflection of the number of transcripts being expressed, we would expect both mutants to show similar defects in synaptic function, distinguished only by the relative severity of the defect. However, the two *nemy* excisions were not merely quantitatively different to each other, they exhibited unique phenotypes. Transmitter release probability was reduced and synaptic plasticity was enhanced in *nemy* ^26.2^ mutants, while the opposite was true for *nemy* ^45^ mutants. PHM function and thus neuropeptide amidation was impaired in *nemy* ^45^ mutants, but not affected in *nemy* ^26.2^ mutants, and finally, defects in *nemy* ^26.2^ mutants could be rescued by feeding octopamine to larvae, while the same feeding protocol had no effect on *nemy* ^45^ mutants. These results suggest that different isoforms of *nemy* have unique functions.

### nemy and TβH function

Octopamine signalling is the invertebrate equivalent of adrenergic signalling in vertebrates. Octopamine receptors are structurally and functionally similar to adrenergic receptors [48,49] and the action of octopamine on these receptors affects a wide array of behaviours [22,24–26]. Even the biosynthesis of octopamine in invertebrates shares similarities with nor-adrenaline biosynthesis in vertebrates. TβH shares 39% identity and 55% similarity with vertebrate Dopamine β hydroxylase [DβH, 22] and converts tyramine to octopamine in the presence of ascorbic acid [10]. Octopamine has been shown to enhance transmitter release and muscle contraction in invertebrates including at the *Drosophila* NMJ [27,50] and has also been shown to affect synaptic outgrowth during starvation [27,28]. The results of the present study suggest that the shorter splice forms of *nemy* may indirectly regulate TβH function.

Previous analysis of *nemy* expression has shown strong localisation of *nemy* in several cells along the midline of the ventral nerve cord [8]. This staining pattern is consistent with reports of TβH expression [22]. In the present study, we showed reductions in transmitter release in *nemy* ^26.2^ mutants similar to those observed in TβH mutants [27]. Furthermore, we were able to rescue the defects in synaptic function by feeding larvae on a diet containing 10 mg/ml octopamine for 24–36 hr prior to experiments. These results suggest that the defects in synaptic function observed in *nemy* ^26.2^ mutants are due to a decrease in TβH function. Interestingly, we did not see any change in transmitter release in *nemy* ^45^ mutants following octopamine feeding suggesting that TβH function, and thus octopamine levels, may not be significantly altered in the *nemy* ^45^ mutants [27,28].

It is worth noting that rescue of synaptic function in *nemy* ^26.2^ mutants on an octopamine supplemented diet cannot be attributed to an acute action of octopamine on its receptors. Muscles 6 and 7 are not directly innervated by octopaminergic neurons [27,51,52], such that octopaminergic modulation of these synapses must occur by paracrine or endocrine release of octopamine from neighbouring muscles or the CNS. Given the relatively large volume of a physiology chamber (1–2 ml), such distant sources of octopamine are unlikely to be effective in an *in situ* assay of synaptic function. As such the rescue of *nemy* ^26.2^ mutants on octopamine supplemented food is indicative of a lasting effect of octopamine released into the haemolymph while the larvae are still intact and may indicate a persistent requirement for octopamine to maintain normal synaptic function.

Another interesting observation of the present study is the role of octopamine in short-term synaptic plasticity. We observed an enhancement of paired pulse plasticity in *nemy* ^26.2^ mutants that was rescued by addition of octopamine to the diet suggesting a role for octopamine in the regulation of paired pulse plasticity. Interestingly however, paired pulse plasticity did not show any significant impairment in TβH mutants, which lack octopamine. One possible explanation for these results may be the presence of multiple octopamine receptors at the NMJ. Recent work examining the role of octopamine in starvation induced synaptic growth has shown that octopamine induces synaptic outgrowth by activation of Octβ2R receptors [27], but also impairs synaptic outgrowth via an action on Octβ1R receptors [28]. It is possible that octopamine may exert similarly antagonistic effects on synaptic function through these receptors. In the absence of any octopaminergic signalling, as seen in TβH mutants, the net effect results in no significant change in paired pulse plasticity. In *nemy* ^26.2^ mutants however, TβH function may be impaired but not entirely absent, since *nemy* ^26.2^ mutants do not show defects in egg laying such as those seen in TβH mutants [22]. In these mutants, the loss or reduction of octopamine from selected sources leads to an enhancement of paired pulse plasticity that can be rescued by feeding octopamine to the larvae.

### *nemy* and PHM function

Neuropeptides regulate a diverse array of biological processes through their actions as neuromodulators and endocrine hormones. All neuropeptides are synthesized as part of a larger, inactive neuropeptide precursor protein, which is post-translationally modified to generate one or more active neuropeptides [7]. The most common form of post-translational modification of neuropeptide precursors in *Drosophila* is C-terminal amidation of glycine-extended peptides by the combined actions of PHM and PAL (peptidyl-glycine α-lyase) [12]. It has been suggested that as many as 90% of all neuropeptides in *Drosophila* undergo C-terminal amidation [13]. PHM catalyzes the first and rate-limiting step of this C-terminal amidation, and is thought to be a key regulatory point in the processing of neuropeptides [11]. PHM activity has been directly linked to the regulation of several behaviours and biological processes including developmental transitions during embryonic and larval development [13] and circadian locomotor rhythms [14]. In addition, specific amidated neuropeptides such as the pituitary adenylate cyclise peptide (PACAP) and the FMRF family of neuropeptides have also been shown to affect behaviours including sleep maintenance [15], learning and memory [16–18] and synaptic function and plasticity [19–21].

The results of the present study confirm our previous observation of reduced neuropeptide amidation in *nemy* ^45^ mutants [8]. This decrease in peptide amidation is likely due to a decrease in PHM function. It should be noted however that *nemy* ^45^ mutants do not completely abolish PHM function. Homozygous PHM mutants die as late embryos or early larvae [13] while *nemy* ^45^ mutants are completely viable. It is possible that the remaining *nemy* isoforms also affect PHM function. While we did not observe a difference in the amidation of FMRF peptides in *nemy* ^26.2^ mutants, we cannot rule out a role for shorter *nemy* isoforms in the amidation of other neuropeptides. We have previously compared the expression of *nemy* and PHM in the larval CNS, and while there is considerable overlap in the expression patterns, not all PHM expressing cells showed expression of *nemy* [8]. Recent work identified a second cytochrome b561 homologue in *Drosophila* that is regulated by the transcription factor DIMMED [53]. DIMMED has been shown to be the primary transcriptional regulator of peptidergic cell fate in *Drosophila* [54]. Interestingly, while the other cytochrome b561 homologue (current designation CG1275) was upregulated by DIMMED expression along with PHM, the study did not find any increase in *nemy* expression [53]. Combined with the results of the present study, these results suggest that the cytochrome b 561 homologues in *Drosophila* play unique roles in regulating the function of PHM and possibly other mono-oxygenases. It should be noted however that our results may indicate the presence of a feedback loop between the expression/function of the cytochrome b561 homologues. The increased amidation of TPAEDFMRF neuropeptides observed in *nemy* ^26.2^ mutants may be the result of a compensatory increase in the expression of CG1275. While *nemy* is not the only cytochrome 561 homologue associated with peptidergic cells in *Drosophila*, the results of the present study suggest that the longest splice form of *nemy* affects PHM function and is required for the normal amidation of at least a subset of neuropeptides.

An interesting observation from the present study is that both *nemy* ^*45*^ and heterozygous PHM mutants result in elevated neurotransmitter release and decreased plasticity at the NMJ. However, the molecular identity of the neuropeptide(s) responsible for this change in NMJ function remains to be determined. Despite a wealth of knowledge surrounding the roles of specific neuropeptides in specific functions [7], relatively little information exists as to the effects of these peptides on NMJ function. One family of neuropeptides that has been well characterized at the NMJ are FMRFamides. This family of neuropeptides are characterized by the signature C-terminal tetra-peptide sequence Phe-Met-Arg-Phe-NH_2_ [55] and have been shown to synaptic function in both vertebrates and invertebrates [20,21,56,57].

In the present study, we showed a decrease in the levels of amidated FMRF neuropeptides, including the most abundant species (DPKQDFMRFa) in *nemy* ^45^ mutants. Given that acute application of FMRFamide peptides enhances presynaptic calcium influx and transmitter release [21], the reduction of FMRFamide peptides might be expected to reduce transmitter release. However, we observed a significant increase in transmitter release in the *nemy* ^45^ mutants. While we did observe a decrease in transmitter release in the *nemy* ^26.2^ mutants, this is unlikely to be due to changes in the level of FMRFamides. First, MS data showed no significant changes in the levels of amidated neuropeptides in the *nemy* ^26.2^ mutants, and second, the reduction in transmitter release observed in the *nemy* ^26.2^ mutants was completely rescued by feeding octopamine to the larvae, suggesting that this reduction was due to an effect of TβH function and octopamine synthesis. Interestingly, the increase in transmitter release observed in *nemy* ^45^ mutants was strikingly similar to that observed in heterozygous PHM mutants, strengthening the suggestion that these defects are due to a decrease in PHM function. While the identity of the neuropeptide(s) affected in *nemy* ^45^ and PHM heterozygous mutants remains to be determined, the results suggest that a predominant regulatory feature of NMJ function includes an as yet unidentified inhibitory neuropeptide. Further work will be required to uncover the identity of this peptide.

## Conclusion

The results of the present study show isoform specific roles for *nemy* in regulating NMJ function. Taken together, these results suggest that the longest *nemy* splice form, which is reduced in *nemy* ^45^ mutants, affects NMJ function predominantly by a reduction in PHM function. Alternatively, the shorter splice forms of *nemy*, which are reduced in the *nemy* ^26.2^ mutant, appear to affect NMJ function predominantly by a reduction in TβH function. In situ localization data suggests that the specific roles for the *nemy* isoforms may arise from different cellular localizations of the transcripts. While these effects of *nemy* on synaptic function are indirect, the different phenotypes observed in independent *nemy* mutants suggest cytochrome b561 may be an important regulator of neuromodulation. Regulation at this level may be particularly important for the integration and/or synchronization of different neuromodulatory signals. While neuropeptide release normally depends on extracellular calcium entry through voltage-gated channels, a recent study showed that octopamine could stimulate neuropeptide release independently of extracellular calcium at the NMJ [58]. Other recent studies have also observed interesting and novel interactions between the neuromodulatory effects of different biogenic amines [5,59]. While neuromodulation of synaptic strength has been viewed as an important factor in the regulation of behavioural plasticity for some time, these studies, combined with the present study suggest that neuromodulation of behavioural plasticity may be more complex than previously understood.

## Supporting Information

S1 File Materials and Methods. Third instar larvae were dissected in PBS and fixed in 4% paraformaldehyde for 25 min at room temperature, then washed in PBST three times (each 10 min), blocked in blocking solution (PBST with 5% normal goat serum, 1% BSA) for 60 min. Primary antibodies were: guinea-pig anti-Nemy 1:20, rabbit anti-Nemy 1:20, mouse anti-Dlg 1:20 (DSHB), FITC-labeled goat anti-HRP1:250 (The Jackson Laboratory), rabbit anti-GFP 1:200 (Life Technologies). Secondary antibodies were: goat anti-guinea-pig Alexa-Fluor 488 1:300 (Life Technologies), goat anti-rabbit Alexa-Fluor 555 1:300 (Molecular Probes) or goat anti-rabbit Alexa-Flour 488 1:300 (Life Technologies), goat anti-mouse Alexa-Fluor 555 1:300 (Molecular Probes). All antibodies were incubated for 2 h at room temperature or overnight at 4°C. All images were obtained using a Leica DMRA2 fluorescent microscope (Leica, Deerfield, IL). **S1 Fig A. Peptide amidation levels. (A1) Peptide** amidation in control cantonised *w* ^1118^ and *Canton S* lines. NS indicates non-significant difference. (**A2)** Peptide amidation in control *Canton S* and *nemy* mutant lines. * indicates a significant difference (P < 0.05) compared with *w* ^1118^ and # indicates a significant difference (P < 0.05) compared with *nemy* ^26.2^ mutants. **B. Third instar larval NMJs**. Muscles 6/7 were immunostained with guinea-pig #1 (B1)and guinea-pig #2 **(B2**) anti-Nemy. The arrows point to the center of synaptic boutons that express Nemy. **C. Nemy is expressed at NMJs.** Images of third instar NMJs formed on muscles 6 and 7 stained with rabbit anti-Nemy (**C1**), FITC conjugated goat anti-horse radish peroxidase (**C2**) and its co-localization (**C3**). The arrows point to the center of synaptic boutons that express Nemy. **D**. **Nemy expression at NMJs can be detected using Nemy-GAL4.** (**D1**) Double staining for *nemy* ^335^GAL4:10XUAS-mCD8-GFP at the larvae NMJ, muscles 6/7. GFP fluorescence pattern, anti-DLG immunereactivity (**D2**) and merged red and green channels (**D3**).
(DOCX)
