# Supplementary material for: Distinct Regulation of Transmitter Release at the Drosophila NMJ by Different Isoforms of nemy
Source: PLoS One. 2015 Aug 3;10(8):e0132548. doi: 10.1371/journal.pone.0132548 (PMC4523183; doi:10.1371/journal.pone.0132548)
Supplement: S1 File — Third instar larvae were dissected in PBS and fixed in 4% paraformaldehyde for 25 min at room temperature, then washed in PBST three times (each 10 min), blocked in blocking solution (PBST with 5% normal goat serum, 1% BSA) for 60 min. Primary antibodies were: guinea-pig anti-Nemy 1:20, rabbit anti-Nemy 1:20, mouse anti-Dlg 1:20 (DSHB), FITC-labeled goat anti-HRP1:250 (The Jackson Laboratory), rabbit anti-GFP 1:200 (Life Technologies). Secondary antibodies were: goat anti-guinea-pig Alexa-Fluor 488 1:300 (Life Technologies), goat anti-rabbit Alexa-Fluor 555 1:300 (Molecular Probes) or goat anti-rabbit Alexa-Flour 488 1:300 (Life Technologies), goat anti-mouse Alexa-Fluor 555 1:300 (Molecular Probes). All antibodies were incubated for 2 h at room temperature or overnight at 4°C. All images were obtained using a Leica DMRA2 fluorescent microscope (Leica, Deerfield, IL). S1 Fig A. Peptide amidation levels. (A1) Peptide amidation in control cantonised w 1118 and Canton S lines. NS indicates non-significant difference. (A2) Peptide amidation in control Canton S and nemy mutant lines. * indicates a significant difference (P < 0.05) compared with w 1118 and # indicates a significant difference (P < 0.05) compared with nemy 26.2 mutants. B. Third instar larval NMJs. Muscles 6/7 were immunostained with guinea-pig #1 (B1)and guinea-pig #2 (B2) anti-Nemy. The arrows point to the center of synaptic boutons that express Nemy. C. Nemy is expressed at NMJs. Images of third instar NMJs formed on muscles 6 and 7 stained with rabbit anti-Nemy (C1), FITC conjugated goat anti-horse radish peroxidase (C2) and its co-localization (C3). The arrows point to the center of synaptic boutons that express Nemy. D. Nemy expression at NMJs can be detected using Nemy-GAL4. (D1) Double staining for nemy 335GAL4:10XUAS-mCD8-GFP at the larvae NMJ, muscles 6/7. GFP fluorescence pattern, anti-DLG immunereactivity (D2) and merged red and green channels (D3). (DOCX) [file pone.0132548.s001.docx]

**S1 File**

**Supporting Information**

**Materials and Methods**

Third instar larvae were dissected in PBS and fixed in 4% paraformaldehyde for 25 min at room temperature, then washed in PBST three times (each 10 min), blocked in blocking solution (PBST with 5% normal goat serum, 1% BSA) for 60 min. Primary antibodies were: guinea-pig anti-Nemy 1:20, rabbit anti-Nemy 1:20, mouse anti-Dlg 1:20 (DSHB), FITC-labeled goat anti-HRP1:250 (The Jackson Laboratory), rabbit anti-GFP 1:200 (Life Technologies). Secondary antibodies were: goat anti-guinea-pig Alexa-Fluor 488 1:300 (Life Technologies), goat anti-rabbit Alexa-Fluor 555 1:300 (Molecular Probes) or goat anti-rabbit Alexa-Flour 488 1:300 (Life Technologies), goat anti-mouse Alexa-Fluor 555 1:300 (Molecular Probes). All antibodies were incubated for 2 h at room temperature or overnight at 4°C. All images were obtained using a Leica DMRA2 fluorescent microscope (Leica, Deerfield, IL).

**
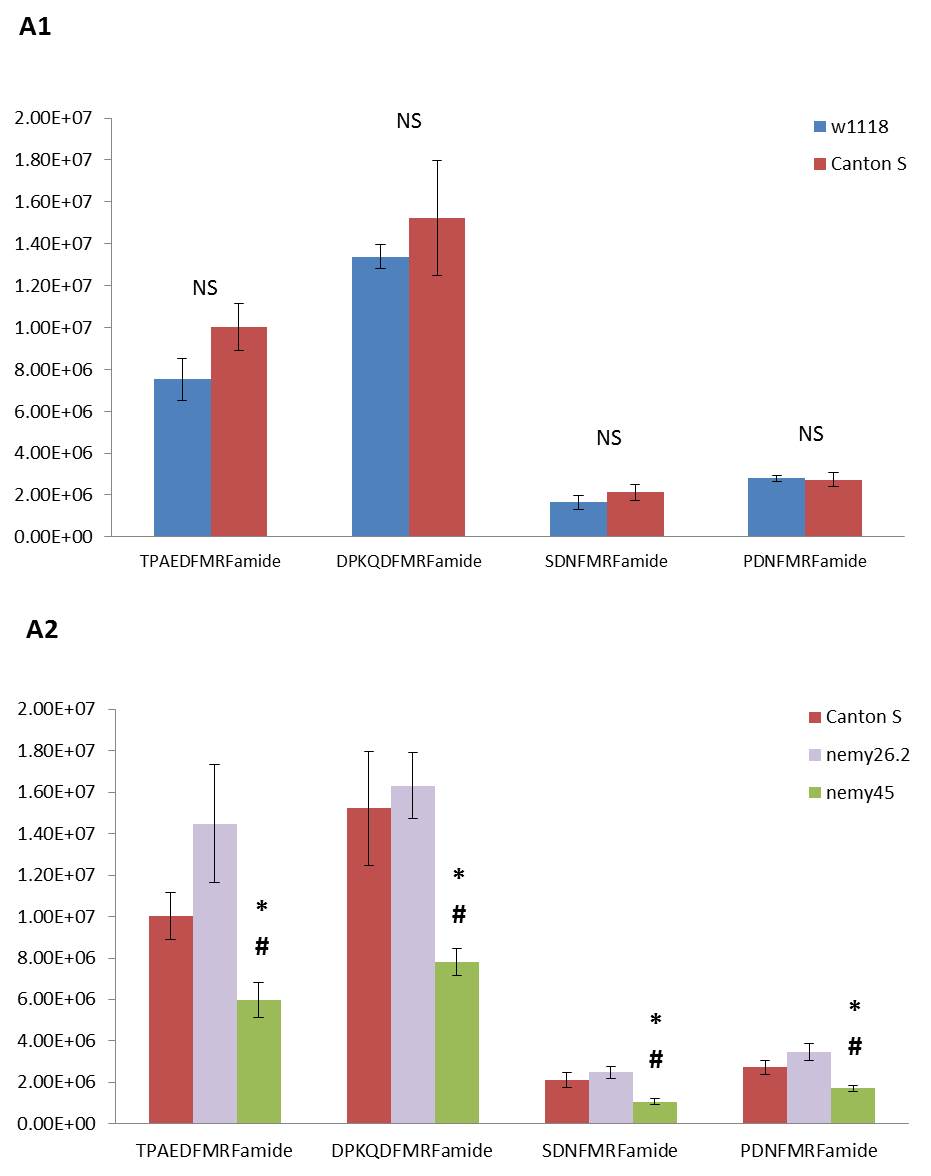
**

**S1 Fig. A Peptide amidation levels. (A1)** Peptide amidation in control cantonised *w*^1118^ and *Canton S* lines. NS indicates non-significant difference. **(A2)** Peptide amidation in control *Canton S* and *nemy* mutant lines. * indicates a significant difference (P < 0.05) compared with *w*^1118^ and # indicates a significant difference (P < 0.05) compared with *nemy*^26.2^ mutants.


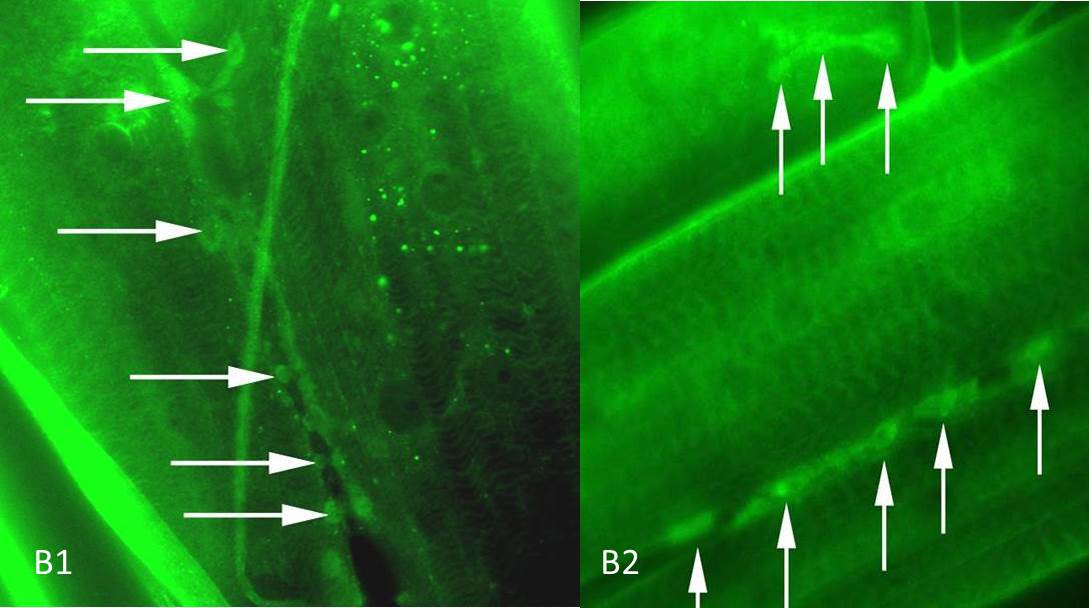


**S1 Fig. B. Third instar larval NMJs**. Muscles 6/7 were immunostained with guinea-pig #1 (**B1**) and guinea-pig #2 (**B2)** anti-Nemy. The arrows point to the center of synaptic boutons that express Nemy.

**
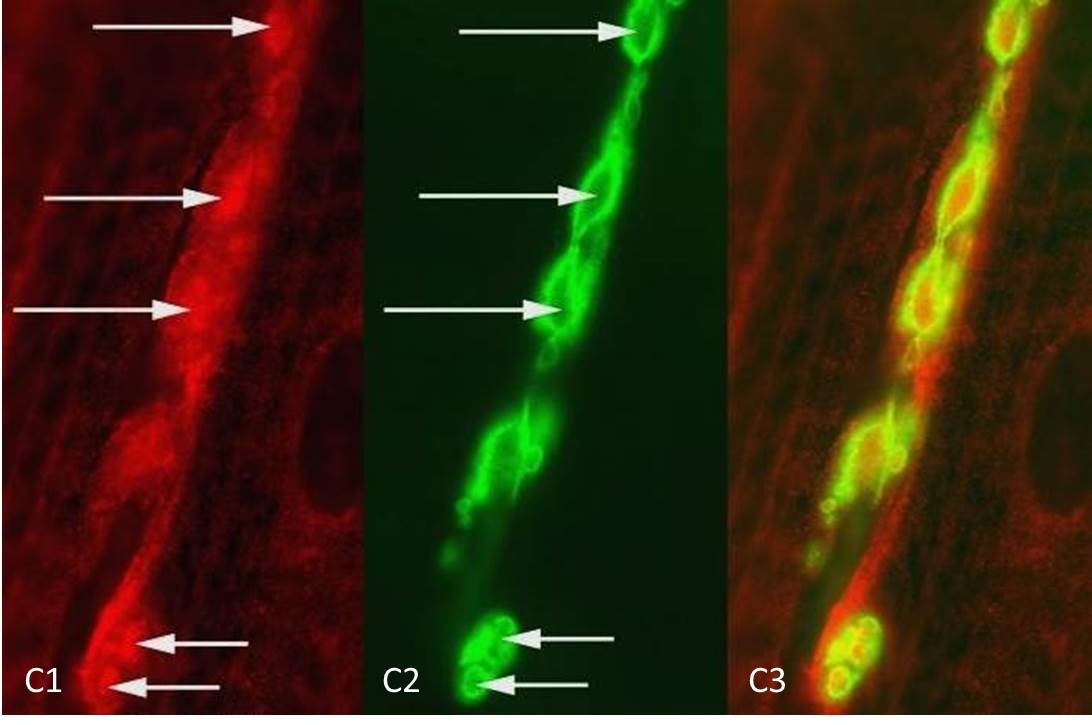
**

**S1 Fig C.** **Nemy is expressed at NMJs.** Images of third instar NMJs formed on muscles 6 and 7 stained with rabbit anti-Nemy (**C1**), FITC conjugated goat anti-horse radish peroxidase (**C2**) and its co-localization (**C3**). The arrows point to the center of synaptic boutons that express Nemy.

**
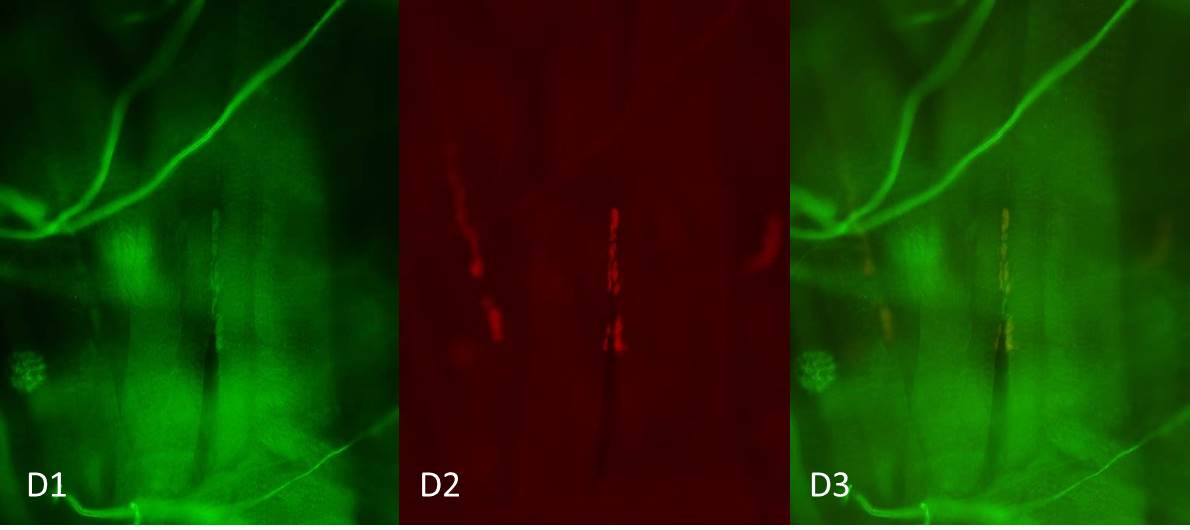
**

**S1 Fig. D.** **Nemy expression at NMJs can be detected using Nemy-GAL4.** (**D1**)Double staining for *nemy*^335^GAL4:10XUAS-mCD8-GFP at the larvae NMJ, muscles 6/7. GFP fluorescence pattern, anti-DLG immunereactivity (**D2**) and merged red and green channels (**D3**).
